# Asymmetric Interaction of Neuropeptidase Activities between Cortico-Limbic Structures, Plasma and Cardiovascular Function after Unilateral Dopamine Depletions of the Nigrostriatal System

**DOI:** 10.3390/biomedicines10020326

**Published:** 2022-01-29

**Authors:** Inmaculada Banegas, Isabel Prieto, Ana Belén Segarra, Francisco Vives, Magdalena Martínez-Cañamero, Raquel Durán, Juan de Dios Luna, Marc de Gasparo, Germán Domínguez-Vías, Manuel Ramírez-Sánchez

**Affiliations:** 1 Department of Health Sciences, University of Jaén, 23071 Jaen, Spain; ibanegas@ujaen.es (I.B.); iprieto@ujaen.es (I.P.); asegarra@ujaen.es (A.B.S.); canamero@ujaen.es (M.M.-C.); 2 Department of Physiology, Faculty of Health Sciences, University of Granada, 18071 Granada, Spain; rduran@ugr.es (R.D.); fvives@ugr.es (F.V.); germandv@go.ugr.es (G.D.-V.); 3 Department of Biostatistic, University of Granada, 18071 Granada, Spain; jdluna@ugr.es; 4 Cardiovascular & Metabolic Syndrome Adviser, Rue es Planches 5, 2842 Rossemaison, Switzerland; m.de_gasparo@bluewin.ch

**Keywords:** neuropeptides, neuropeptidases, neurochemical asymmetry, functional asymmetry, neurovisceral integration

## Abstract

In emotional processing, dopamine (DA) plays an essential role, and its deterioration involves important consequences. Under physiological conditions, dopamine exhibits brain asymmetry and coexists with various neuropeptides that can coordinate the processing of brain functions. Brain asymmetry can extend into a broader concept of asymmetric neurovisceral integration, including behavior. The study of the activity of neuropeptide regulatory enzymes (neuropeptidases, NPs) is illustrative. We have observed that the left and right brain areas interact intra- and inter-hemispherically, as well as with peripheral tissues or with physiological parameters such as blood pressure or with behaviors such as turning preference. To obtain data that reflect this integrative behavior, we simultaneously analyzed the impact of left or right brain DA depletion on the activity of various NPs in corticolimbic regions of the left and right hemispheres, such as the medial prefrontal cortex, amygdala and hippocampus, as well as on the plasma activity of the same aminopeptidase activities (APs) and on systolic blood pressure (SBP). Intra- and inter-hemispheric interactions as well as the interactions of NPs from the left or right hemispheres were analyzed with the same plasma APs and the SBP obtained from sham and from left or right lesioned rats. The results demonstrate a complex profile depending on the hemisphere considered. They definitively confirm an asymmetric neurovisceral integration and reveal a higher level of inter-hemispheric corticolimbic interactions including with SBP after left dopamine depletion.

## 1. Introduction

The 1970s were a turning point in brain research: the work of Pert and Snyder [1] and of Hughes and Kosterlitz [2] unleashed a revolution in which new peptide neurotransmitters called neuropeptides were discovered [3]. The analysis of the brain’s functional regulation of neuropeptides was in part carried out through the study of its proteolytic processing [4] as a result of the action of neuropeptidases (NPs) [5,6].

In emotional processing, dopamine (DA) plays an essential role: its deterioration, for example in Parkinson’s disease, may involve important emotional consequences that implicate, more particularly, corticolimbic regions such as the medial prefrontal cortex (mPFC), hippocampus (HC) or the amygdala (AM) [7,8]. Dopamine coexists and interacts with other neurotransmitters, including various neuropeptides [9] that may participate in the pathogenesis of classic disorders that implicate DA (such as Parkinson’s disease and others). Under physiological conditions, DA shows an inter-hemispheric asymmetry [10], which is also obvious in pathological processes such as the first stages of development in Parkinson’s disease [11]. However, the response must be comprehensive, as it involves not only the unilateral intra-hemispheric interaction between corticolimbic structures, but also the inter-hemispheric interaction between such structures, as well as the correlation of all of them with plasma elements or with peripheral functions such as cardiac function, as recently reviewed [12]. In particular, the analysis of neuropeptidases and some biochemical factors in the brain as a whole or considering left and right brain hemispheres, in peripheral tissues and fluids together with cardiovascular or renal physiological functions, demonstrated asymmetrical effects and interactions between them under different experimental conditions. For example, captopril treatment changed the left predominance of correlations between neuropeptidases of the frontal cortex and aminopeptidases of ventricular tissue observed in untreated animals, and to the right in the treated ones. Genetically hypertensive rats radically increased blood pressure levels after DA depletion of the left hemisphere with 6-hydroxydopamine (6-OHDA) but not by depletion of the right. Additionally, there was a left predominance of correlations between frontal cortex and blood pressure, heart rate or water balance functions. Although correlation is not causation, all these results (and others) are suggestive of a dynamic brain asymmetry that we can extend to an asymmetric neurovisceral integration (reviewed in [12]). The present work essentially analyzes a possible pattern of asymmetrical neurovisceral interaction that involves intra- and inter-hemispheric interactions between some corticolimbic structures and between such structures and systolic blood pressure (SBP), as a peripheral functional parameter, and plasma aminopeptidase activities in controls and left or right DA-depleted animals. 

Some limited data have been provided on the asymmetric distribution of various neuropeptidases such as oxytocinase [13], enkephalinase [14] or neuropeptidases hydrolyzing angiotensins and cholecystokinin [15] in specific regions similar to the ones analyzed in the present work, such as prefrontal cortex of animals unilaterally depleted of DA. However, an integrative neurovisceral interaction such as the simultaneous intra- and inter-hemispheric interaction of these activities has not yet been analyzed, neither between these activities of the left or right hemispheres versus plasma or between them and cardiac function. 

In order to obtain data that help us to understand this integral functioning of the organism, we simultaneously analyzed the effect of left or right brain DA depletion on the activity of various NPs such as alanyl-(AlaAP), cystinyl-(CysAP), glutamyl-(GluAP) and aspartyl-aminopeptidase (AspAP) reported as hydrolyzing NPs, respectively, of enkephalins, oxytocin, angiotensin and cholecystokinin peptides [13,14,15], in corticolimbic regions of the left and right hemispheres, such as the mPFC, AM and HC, as well as on the plasma activity of the same aminopeptidase activities (APs) and on SBP. Intra- and inter-hemispheric interactions are analyzed, as well as the interactions of NPs from the left or right hemispheres with the same plasma APs and the SBP obtained from left or right sham animals and from left or right lesioned animals.

## 2. Materials and Methods

### 2.1. Experimental Design

**Figure 1:**
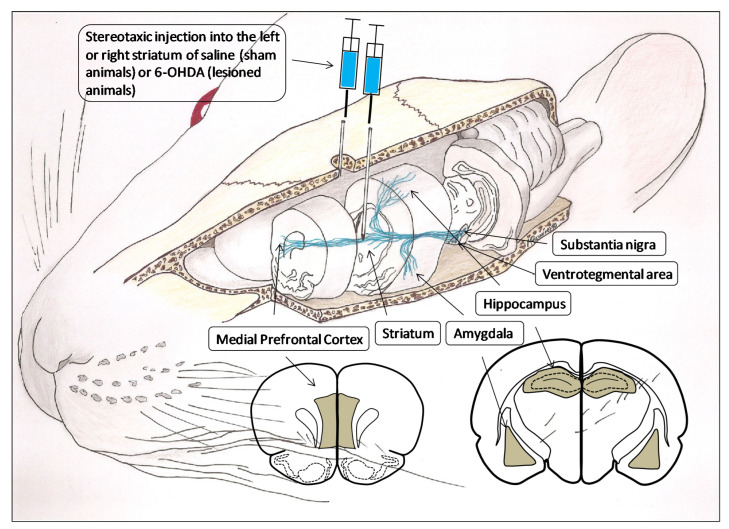
Three-dimensional representation of the brain dopaminergic system in which the areas involved in the present investigation are considered: the substantia nigra and ventrosegmental area, as origin of dopaminergic pathways and their projection to the striatum, amygdala, hippocampus and medial prefrontal cortex. Likewise, the figure also includes the anterior face of the slices from which the analyzed left and right brain areas were obtained (gray areas). The stereotaxic injection of saline or 6-hydroxydopamine (6-OHDA) into the left or right striatum is performed to generate, respectively, left or right control groups (sham operated) or left or right lesioned groups.

**Figure 2:**
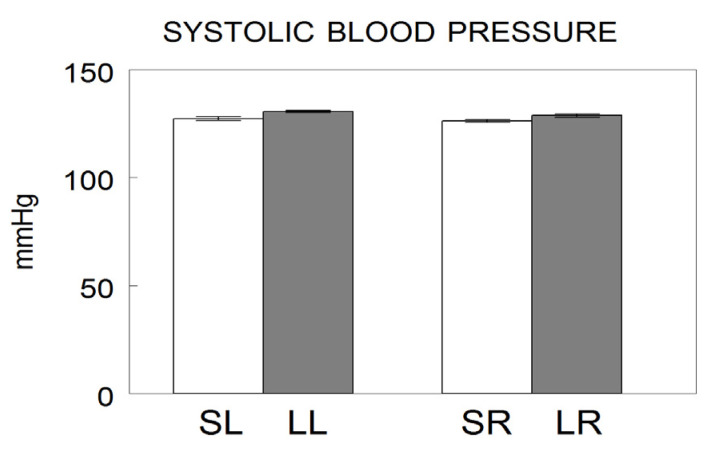
Systolic blood pressure values after saline injection into the left (Sham left; SL) or right (Sham right; SR) or 6-hydroxydopamine into the left (Lesioned left; LL) or right (Lesioned right; LR) striatum of Wistar-Kyoto adult male rats. The values represent the mean ± SEM of the SBP expressed in mmHg (modified from [17]).

A total of 40 adult male Wistar-Kyoto (WKY) rats (Charles River Laboratories, Barcelona, Spain), divided into four groups of 10 each, were used in this study. Depending on the hemisphere considered, the groups were: sham left (SL), sham right (SR), lesioned left (LL) and lesioned right (LR). The degeneration of the left or right nigrostriatal dopaminergic pathway, and consequently, the left or right hemispheric depletion of dopamine, was carried out using the neurotoxin 6-OHDA injected stereotaxically into the left or right striatum [16]. Sham groups were obtained by the left or right injection of saline into the striatum (Figure 1). The animals that were lesioned demonstrated, four weeks after 6-OHDA injections, a striking turning behavior towards the hemisphere with less dopamine content [15]. At the end of the experimental period, systolic blood pressure (SBP) was measured by plethysmographic method as previously reported in detail [17] (Figure 2). The rats were then sacrificed on the same day and blood samples as well as samples from the left or right medial prefrontal cortex (mPFC), hippocampus (HC) and amygdala (AM) were obtained from each group and frozen until assayed. All the experiments and the care of animals were performed in agreement with the European Communities Council Directive 86/609/EEC. Approved by the bioethic committee of the University of Jaén on 20 November 2006 with number 140. 

### 2.2. 6-Hydroxydopamine and Saline Intrastriatal Injections

Under equithensin (42.5 g/L chloral hydrate dissolved in 19.76 mL ethanol, 9.72 g/L Nembutal^®^, 0.396 g/L propylenglycol and 21.3 g/L magnesium sulfate in distilled water) anesthesia (2 mL/kg body weight), the animals were positioned stereotaxically (David Kopf Instruments, Palo Alto, CA, USA). A 2 mm burr hole was drilled just through the skull at horizontal coordinates approximating the position of the striatum, that is, AP 0 mm, L or R 3 mm and H-5 mm, according to the stereotaxic atlas of Paxinos and Watson [18]. For the groups of lesioned animals, 4 μL of 6-OHDA (8 mg dissolved in 1 mL of cold saline with 0.02% ascorbic acid to inhibit oxidation) was administered into the left or right striatum. Sham groups were handled in the same way, but, instead of 6-OHDA, they received 4 μL of saline with 0.02% ascorbic acid. (Figure 1). The injection of 6-OHDA into the striatum was selected as the most appropriate in order to lesion only neurons of the nigrostriatal pathway and perform the unilateral depletions of DA [16,19].

### 2.3. Obtaining Blood and Brain Samples

Blood samples were obtained through the left cardiac ventricle under equithensin anesthesia four weeks after intrastriatal injections of 6-OHDA or saline, once the turning motor behavior was determined [15]. Plasma was obtained by centrifugation for 10 min at 3000× *g* and used to determinate aminopeptidase activities and proteins in triplicate. After blood sample collection, the rats were perfused with saline through the left cardiac ventricle and the brain was quickly removed (less than 60 s) and cooled on dry ice. Left and right samples of the mPFC, HC and AM were dissected according to the stereotaxic atlas of Paxinos and Watson [18]. The mPFC was dissected between 12.70 mm and 11.20 anterior to the interaural line (AIL), the HC between 7.12 and 5.40 mm AIL, and the AM between 7.12 and 5.40 mm AIL.

### 2.4. Enzymatic and Protein Determinations

The procedures for enzymatic and protein determinations were previously described in detail [6]. Briefly, brain samples were defrosted and homogenized in 400 μL of 10 mM HCl–Tris buffer (pH 7.4) and ultracentrifuged at 100,000× *g* for 30min at 4 °C. To solubilize membrane proteins, the pellets were re-homogenized in HCl–Tris buffer (pH 7.4) plus 1% Triton-X-100. After centrifugation (100,000× *g*, 30 min, 4 °C), the supernatants were shaken in an orbital rotor for 2 h at 4 °C with the polymeric adsorbent Bio-Beads SM-2 (100 mg/mL) to remove the detergent from the sample. After the biobeads’ removal, the supernatants were used to measure membrane-bound neuropeptidase activities (NPs) and protein content in triplicate. Neuropeptidase activities, in the selected brain regions, were fluorometrically measured using aminoacyl-naphthylamide (aaNNap) as substrates, as previously described [6]. Briefly, AlaAP and CysAP were fluorometrically determined, using AlaNNap and CysNNap as substrates; 10 μL of each supernatant was incubated for 30 min at 25 °C with 1 mL of the substrate solution: 2.14 mg/100 mL AlaNNap or 5.63 mg/100 mL CysNNap, 10 mg/100 mL bovine serum albumin (BSA), and 10 mg/100 mL dithiothreitol (DTT) in 50mM of phosphate buffer, pH 7.4, for AlaAP; and 50 mM HCl–Tris buffer, pH 6, for CysAP. AspAP was determined using AspNNap as substrate: 10μL of each supernatant was incubated for 120 min at 37 °C with 1 mL of the substrate solution (2.58 mg/100 mL AspNNap, 10 mg/100 mL BSA and 39.4 mg/100 mL MnCl_2_ in 50 mmol/L HCl–Tris buffer, pH 7.4). GluAP was measured using GluNNap as substrate: 10 μL of each supernatant was incubated for 120 min at 37 °C with 1 mL of the substrate solution (2.72 mg/100 mL GluNNap, 10 mg/100 mL BSA, 10 mg/100 mL DTTand 0.555 g/100 mL CaCl_2_ in 50 mmol/L HCl–Tris, pH 7.4). To stop enzymatic reactions, 1 mL of 0.1 mol/L of acetate buffer, pH 4.2, was added. The amount of β-naphthylamine (released as a consequence of enzymatic activity) was measured fluorometrically at 412 nm emission wavelengths with an excitation wavelength of 345 nm. The sensitivity of the method allows for measurements of enzymatic activities in the pmolar range. Proteins were quantified in triplicate by the method of Bradford [20], using BSA as a standard. Specific membrane-bound aminopeptidase activities were expressed as nmol of AlaNNap, CysNNap, AspNNap, or GluNNap hydrolyzed per min per mg of protein. Fluorogenic assays were linear with respect to time of hydrolysis and protein content.

### 2.5. Statistical Analysis

**Figure 3:**
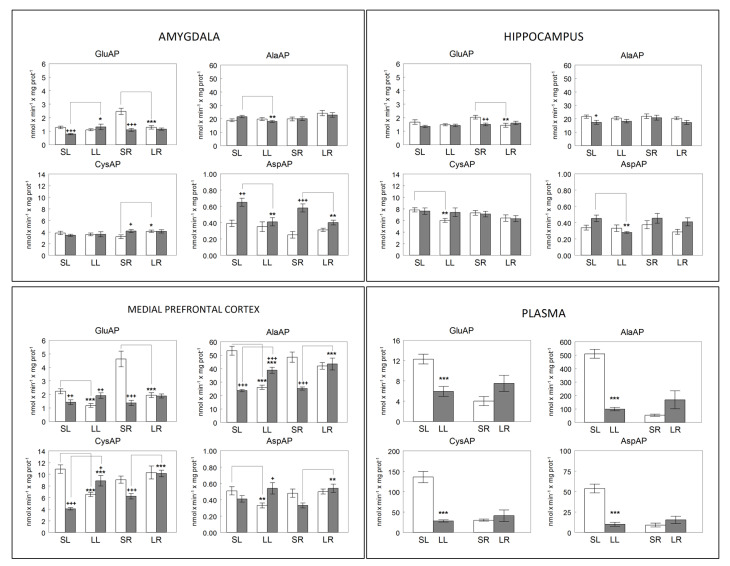
Membrane-bound glutamyl-(GluAP), alanyl-(AlaAP), cystinyl-(CysAP) and aspartyl-(AspAP) aminopeptidase activities expressed, respectively, as nmols of L-Glu-β-naphthylamide, L-Ala-β-naphthylamide, L-Cys-β-naphthylamide and L-Asp-β-naphthylamide hydrolyzed per min per mg of proteins in the left (open bars) or right (gray bars) side of the amygdala, hippocampus, medial prefrontal cortex and plasma of sham left (SL) or sham right (SR) and lesioned left (LL) or lesioned right (LR) adult male normotensive Wistar-Kyoto rats. In plasma, unfilled bars represent sham groups (SL, SR) and gray bars represent lesioned groups (LL, LR). Each group represents the mean ± SEM values for 10 animals. (*) level of statistical significance between the left and right side in brain or between sham vs. lesioned animals in plasma. (+) statistical difference, in the same side, between sham vs. lesioned rats. * (*p* < 0.05), ** (*p* < 0.01) or *** (*p* < 0.001). + (*p* < 0.05), ++ (*p* < 0.01) or +++ (*p* < 0.001). Preliminary data of AlaAP from mPFC [14], CysAP from mPFC [13] and GluAP from plasma [17] and mPFC [15] were previously reported.

To study the intra- and inter-hemispheric levels of correlation between NPs of the mPFC, HC and AM, between them and plasma APs and between them and SBP, Pearson’s coefficient of correlation was computed. Computations were performed using SPSS 13.0 and STATA 9.0. *p*-values below 0.05 were considered significant. Comparisons between means in the different locations (Figure 3) are included for informative purposes but they are not discussed. Some results have already been previously reported and evaluated using two-way analysis of variance [13,14,15,17]. The present work is essentially aimed toward a simultaneous correlation analysis between the various central and peripheral locations and SBP values, in the different groups, as reported below.

## 3. Results

### 3.1. Systolic Blood Pressure

Systolic blood pressure in left or right lesioned animals does not differ from the pressure observed in their corresponding left or right sham controls (Figure 2).

### 3.2. Brain Neuropeptidase and Plasma Aminopeptidase Activities

The bilateral behavior of neuropeptidase activities differs depending on the brain location and the enzyme studied. In plasma, the behavior is similar for all the activities analyzed. A significant decrease is observed after the left lesion in comparison with their corresponding left sham controls (*p* < 0.001). However, there is a tendency to increase after the right lesion, which does not reach statistical significance (Figure 3).

The most significant results indicate that an increase in GluAP is observed in the amygdala on the right side after the left lesion and a decrease on the left side after the right lesion. However, AspAP decreases on the right side in both the left and right lesions. 

In mPFC, AlaAP and CysAP have a similar behavior: they decrease both on the left side and increase on the right after the left lesion and increase only on the right side after the right lesion. GluAP decreases on the left side in both the left and right lesions and does not vary on the right side, whereas AspAP decreases on the left side after the left lesion and increases on the right side after the right lesion.

### 3.3. Sham Left Correlations

In the group of SL, only positive correlations between NPs of the different regions analyzed were observed in the left hemisphere. In contrast, only negative correlations between plasmatic APs and left hemisphere NPs were observed in this group. Moreover, only one negative correlation between CysAP of the left amygdala and SBP (*p* < 0.05) was observed.

There were diverse positive and negative correlations between NPs in the right hemisphere. No correlations were observed between plasma APs and right hemisphere NPs but there were significant correlations between SBP and NPs of the right hemisphere.

**Figure 4:**
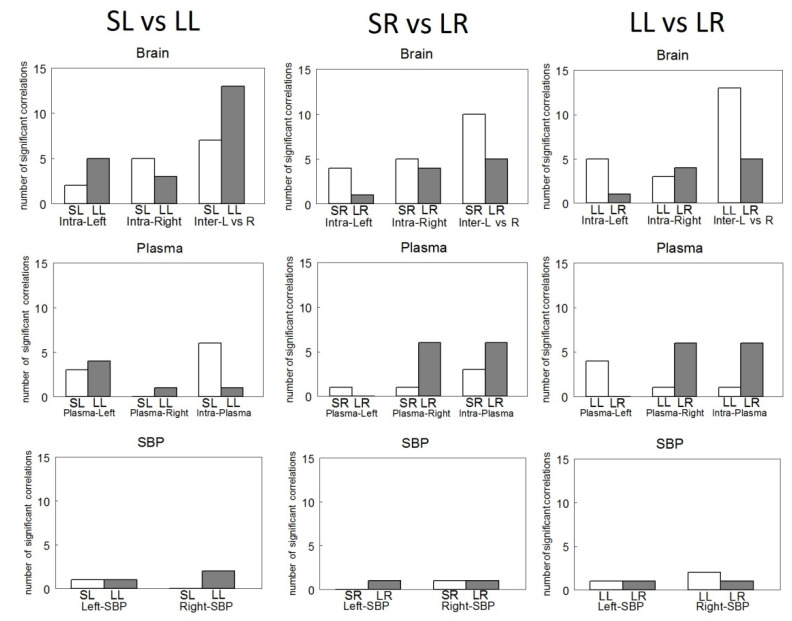
Number of significant intra- (Intra-Left or Intra-Right) and inter-hemispheric (Inter L vs. R) correlations between brain neuropeptidase activities (Brain), between plasma aminopeptidase activities and left (Plasma-Left) or right (Plasma-Right) brain neuropeptidase activities and Intra-Plasma aminopeptidase activities between themselves (Plasma) as well as between left (Left-SBP) or right (Right-SBP) neuropeptidase activities and systolic blood pressure (SBP) in Sham Left (SL), Sham Right (SR), Lesioned Left (LL) or Lesioned Right (LR) WKY rats.

**Figure 5:**
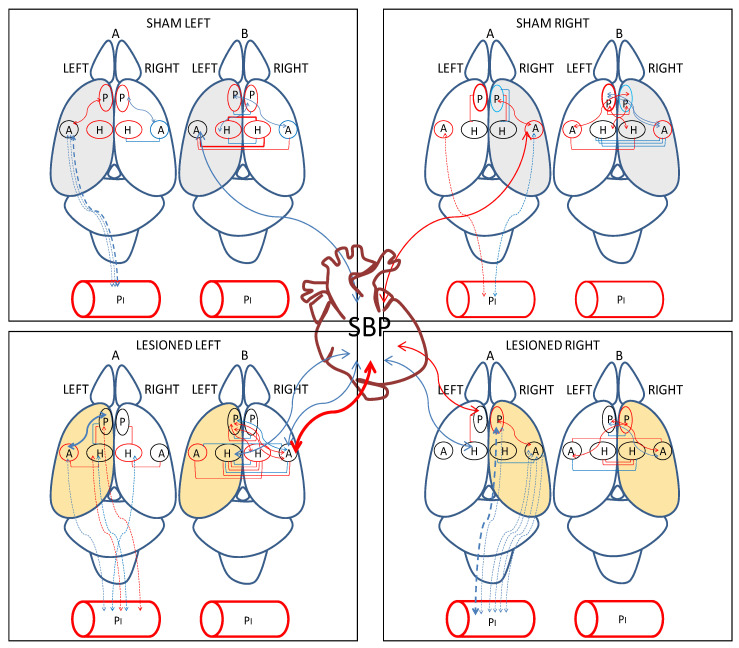
Significant intra- and inter-hemispheric correlations between (a) brain neuropeptidase activities, (b) plasma aminopeptidase activities and brain neuropeptidase activities and (c) brain neuropeptidase activities and systolic blood pressure (SBP) in the four groups studied. Positive correlations in red and negative correlations in blue. Continue lines: significant correlations between brain locations and between brain and SBP. Dotted lines: significant correlations between brain and plasma. The thickness of lines is proportional to the degree of significance. Above rat brain: A, intra-hemispheric correlations, B, inter-hemispheric correlations. The hemisphere injected with saline for sham animals is in grey. The hemisphere injected with 6-hydroxydopamine (6-OHDA) for lesioned animals is in light orange. P, medial prefrontal cortex; A, amygdala; H, hippocampus.

Inter-hemispheric correlations were diverse, positive and negative. However, intra-plasmatic correlations between APs were only positive (Table 1, Figure 4 and Figure 5).

### 3.4. Sham Right Correlations 

Sham right animals also demonstrated only positive correlations between NPs in the left hemisphere and only one positive correlation was observed between plasma CysAP and AlaAP activity of the left amygdala (*p* < 0.05). No correlations were observed between NPs of the left hemisphere and SBP.

Intra-hemispheric correlations in the right hemisphere (as occurred in the right one in SL) were also diverse, positive as well negative. Plasma APs vs. NPs of the right hemisphere only demonstrated one negative correlation between plasma AlaAP vs. AlaAP of the right amygdala (*p* < 0.05). GluAP of the right amygdala correlated positively with SBP (*p* < 0.05).

As occurred in SL, intra-hemispheric correlations in the right side were variably positive as well as negative. Similar to that occurring in SL, intra-plasmatic correlations were always positive (Table 2, Figure 4 and Figure 5).

### 3.5. Lesioned Left Correlations

In LL animals, the significant left intra-hemispheric correlations were both positive and negative. The significant correlations between plasma APs and NPs from left regions were also positive and negative. CysAP activity from left hippocampus correlated negatively with SBP (*p* < 0.01).

Only positive correlations were observed between NPs in the right hemisphere. Plasma AspAP activity correlated negatively (*p* < 0.05) with AlaAP in the right hippocampus. GluAP activity of the right amygdala correlated negatively (*p* < 0.05) with SBP levels. A highly significant correlation (*p* < 0.001) was also observed between CysAP activity of the right amygdala and SBP levels.

In the present LL group, a high number of inter-hemispheric correlations, both positive and negative, were observed, but only a positive intra-plasmatic correlation (*p* < 0.01) was observed between GluAP and CysAP (Table 3, Figure 4 and Figure 5).

### 3.6. Lesioned Right Correlations

In the left hemisphere of LL GluAP demonstrated a significant positive correlation (*p* < 0.05) between GluAP of the mPFC and AlaAP of the hippocampus. No correlations between plasma APs and NPs of left hemisphere were observed. Similar to LL, CysAP of the left hippocampus correlated significantly and negatively (*p* < 0.05) with SBP.

In the right hemisphere, a higher number of significant correlations, both positive and negative, were observed compared to the left hemisphere. In contrast to what happened in the left hemisphere, a higher number of correlations, all of them negative, were observed between plasma APs and NPs of the right hemisphere. In addition, AspAP activity from the right mPFC correlated positively (*p* < 0.05) with SBP.

Inter-hemispheric correlations between NPs were also positives and negatives. However, as occurred in the rest of the groups, correlations between plasmatic APs were always positive (Table 4, Figure 4 and Figure 5).

### 3.7. Comparisons between Groups

In the comparisons between groups, the correlations were similar between SL and SR. However, the most significant contrasts occur in the comparison between plasma APs vs. left hemisphere NPs, which exhibited only negative correlations in SL (see Table 1 vs. Table 2).

Compared with SL, in LL, whereas intra-left hemisphere correlations increase, intra-right correlations decrease. Additionally, inter-hemispheric correlations increase and intra-plasma correlations decrease. In contrast, if we compare SR with LR, the intra-left and inter-hemispheric correlations decrease, but the plasmatic APs of SR vs. right hemisphere of LR increase and the intra-plasma of SR vs. the intra-plasma of LR also increase (Figure 4).

Comparing LL vs. LR, there was a higher number of inter-hemispheric correlations in LL than in LR and there was a lower number of intra-plasma correlations in LL than in LR. LL vs. LR comparison demonstrated an inverse behavior in the frequency of intra-hemispheric correlations: the lower LR vs. LL for intra-left, the higher LR vs. LL for intra-right. Additionally, the same inverse behavior was observed between plasma and the left or right hemisphere: the lower in LR than LL in plasma vs. left hemisphere, the higher in LR than LL in plasma vs. right hemisphere.

In LR, the correlations with SBP are similar to those observed in LL, although with a lower level of significance. In the left hemisphere of LR, hippocampal CysAP correlated negatively (*p* < 0.05) with SBP and in the right side of LR, AspAP of mPFC correlated positively (*p* < 0.05) with SBP. In LR, the total number of inter-hemispheric correlations (both positive and negative) is less than in LL. However, the total number of correlations between plasma APs (all positive) is much higher than what we obtained in LL (Figure 4).

In Figure 5, we can observe that the number of intra-hemispheric correlations was always lower than the number of inter-hemispheric ones in all the groups studied. They were higher in the lesioned groups, especially after the left lesion. The intra-plasmatic correlations were always positive and, in contrast to the inter-hemispheric, the number of intra-plasmatic correlations decreased dramatically in LL. The highest number of hemispheric unilateral correlations of NPs with plasma APs was observed in LR with the right hemisphere. Interestingly, they were all negative in contrast to the left side in which they did not appear. Plasma APs correlated mainly with diverse left hemisphere locations in LL, but especially with the right amygdala in LR. In Sham animals, SBP correlated with the amygdala of the saline-injected hemisphere, whereas in lesioned animals, SBP correlated with diverse locations, mainly with the non-lesioned hemisphere (Figure 5).

## 4. Discussion

In emotional processing, dopamine (DA) plays an essential role: its deterioration, for example in Parkinson’s disease, schizophrenia or attention deficit hyperactive disorder [21], can lead to significant emotional consequences that implicate, among others, corticolimbic regions such as medial prefrontal cortex (mPFC), hippocampus (HC) or the amygdala (AM) [7,8]. Under basal physiological conditions, DA shows an inter-hemispheric asymmetry [10], and a possible abnormal brain asymmetry may be part of the pathogenesis of neural disorders involving DA [5,22,23].

Dopamine coexists and interacts with other neurotransmitters, including various neuropeptides such as enkephalins, oxytocin, cholecystokinin or angiotensins, that also may participate in the pathogenesis of the above-mentioned disorders that implicate DA. These neuropeptides are in part regulated by the proteolytic enzymes analyzed in the present investigation.

The most notable results of this research are: (1) Left or right nigrostriatal lesions differentially affect both intra- and inter-hemispheric correlations as well as plasma APs and SBP. (2) It involves significant intra- and inter-hemispheric correlations between corticolimbic regions, mostly in animals lesioned in the left hemisphere: The dynamics of significant interactions increases and diversifies after lesions, especially after the left lesion.

**Figure 6:**
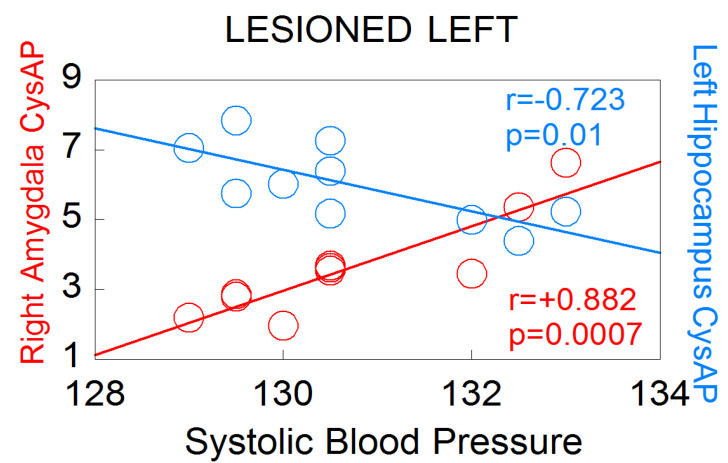
Significant correlations between CysAP of the right amygdala or CysAP of the left hyppocampus, with systolic blood pressure levels in left lesioned animals (n = 10). Red color denotes positive correlation. Blue color indicates negative correlation. Pearson’s correlation coefficients (r) and p values are indicated in the figure.

Particularly noteworthy is the asymmetry observed in the significant correlations between oxytocinase activity (CysAP) in the left or right hemispheres of animals with left lesion and systolic blood pressure (Figure 6). While CysAP in the right amygdala correlates positively with SBP (r = +0.882, *p* = 0.0007) (the higher SBP, the higher CysAP), CysAP in the left hippocampus correlates negatively with SBP (r = −0.723, *p* = 0.01) (the higher SBP, the lower the CysAP, and vice versa). These results demonstrate an inverse behavior in the neurovisceral brain–cardiac interaction in animals with left DA depletion, but not in those with right DA depletion. Interestingly, it has been proposed that part of the actions of oxytocin are due to the fragments of the nonapeptide after the action of oxytokinases such as CysAP [24]. This asymmetrical response suggests important behavioral differences between animals with left or right lesions. The results also indicate differences in cardiovascular consequences depending on the side of the lesion.

The left lesions condition a greater number and level of significance in the inter-hemispheric correlations, in the correlations between NPs with plasma APs and with SBP. Therefore, these results would be in agreement with what was observed in left brain injuries and brain pathologies that affect mainly the left hemisphere in some periods of its evolution [12]. In neurodegenerative disorders such as Parkinson’s disease, Alzheimer’s disease, multiple sclerosis or amyotrophic lateral sclerosis, which manifest brain asymmetry, there is predominantly a greater involvement of the left hemisphere [25]. More severe sensory–motor and cognitive problems were produced after accidents involving the left hemisphere than those produced in the right one [26]. In addition, the brain processing of pain, closely related with emotion-related regions, also exhibits an increase in intra-hemispheric correlations in the left hemisphere [27].

Interestingly, the neuropeptidase activities of the left and right hemispheres (of these same animals) also correlate differentially with the left or right turning behavior of the animal, depending on whether the DA depletion is left or right. [15]. Particularly, in comparison with the rest of groups (SL, SR and LR), there was a predominance of significant correlations between striatum and mPFC and also between the striatum of the left side with the right turning behavior in right lesioned animals [15]. Therefore, the present data together with the previous ones definitely demonstrate an asymmetric neurovisceral response that integrally involves not only visceral processes but also the behavior of the animal in which asymmetric neurochemical modifications, in this case of DA and neuropeptidases, are implicated.

The results support the dynamic character of neurovisceral asymmetry, which involves intracerebral neurochemical correlations: correlations of brain NPs with plasma APs, as well as brain NPs with cardiovascular function, which, together with the existence of significant interactions with motor behavior in these same animals, strongly support the dynamic concept of an asymmetric neurovisceral integration. This implies a neurochemical substrate and suggests that the emotional processes in which the studied areas are involved are also carried out asymmetrically.

In conclusion, the fact that a clear systematic response appears to depend on the side of the lesion supports the concept that we are facing a functionally compensatory physiological response. It remains to be analyzed whether the central and peripheral processes could be related to the changes in NPs, APs and SBP, observed in the present research, after left or right dopamine depletions. These results could be important to understand pathologies with an important asymmetric component such as Parkinson’s disease, schizophrenia, depression, attention deficit hyperactive disorder and others in which a dopaminergic alteration is underlying.

## Figures and Tables

**Table 1:** Significant intra- and inter-hemispheric correlations between brain neuropeptidase activities, between plasma aminopeptidase activities and brain neuropeptidase activities as well as between neuropeptidase activities and SBP in sham left WKY rats.

SHAM LEFT WKY		
Sham Left WKY Left Side	Sham Left WKY Right Side	Sham Left WKY Left Side vs
Brain vs Brain	Brain vs Brain	*LPAla vs RPCys r = −0.654 p = 0.04*
LHAla vs LHCys r = +0.705 p = 0.022	RPGlu vs RPAla r = +0.710 p = 0.021	*LPCys vs RAAla r = −0.679 p = 0.03*
LPAla vs LAAsp r = +0.632 p = 0.049	*RPAsp vs RAGlu r = −0.641 p = 0.045*	LAGlu vs RHCys r = +0.772 p = 0.0089
LPGlu vs LPAsp r = +0.670 p = 0.034	*RAGlu vs RACys r = −0.839 p = 0.0024*	LAAla vs RAAla r = +0.760 p = 0.01
	*RACys vs RHAsp r = −0.644 p = 0.044*	*LHGlu vs LPGlu r = −0.680 p = 0.03*
	RHAla vs RHCys r = +0.725 p = 0.017	LHAla vs RHCys r = +0.827 p = 0.0031
		*LHAsp vs RHCys r = −0.701 p = 0.023*
Plasma vs Brain Left Side	Plasma vs Brain Right Side	Sham Left WKY Plasma
*PlAla vs LAGlu r = −0.789 p = 0.0066*	NO CORRELATIONS	Plasma vs Plasma
*PlCys vs LAGlu r = −0.733 p = 0.0157*		PlGlu vs PlAla r = +0.888 p = 0.0006
*Pl Glu vs LAGlu r = −0.756 p = 0.0113*		PlGlu vs PlCys r = +0.812 p = 0.0043
		PlGlu vs PlAsp r = +0.705 p = 0.0226
Brain Left Side vs SBP	Brain Right Side vs SBP	PlAla vs PlCys r = +0.782 p = 0.0074
*LACys vs SBP r = −0.699 p = 0.02 *	NO CORRELATIONS	PlAla vs PlAsp r = +0.734 p = 0.0155
		PlCys vs PlAsp r = +0.649 p = 0.04

The *p*-values and Pearson’s correlation coefficients (r) are indicated. Significant negative correlations in blue italics. Significant positive correlations in red. (L) left, (R) right, (H) hippocampus, (P) medial prefrontal cortex, (A) amygdala, (Glu) GluAP, (Ala) AlaAP, (Cys) CysAP and (Asp) AspAP.

**Table 2:** Significant intra- and inter-hemispheric correlations between brain neuropeptidase activities, between plasma aminopeptidase activities and brain neuropeptidase activities as well as between neuropeptidase activities and SBP in sham right WKY rats.

SHAM RIGHT WKY		
Sham Right WKY Left Side	Sham Right WKY Right Side	Sham Right WKY Left Side vs
		Right Side
Brain vs Brain	Brain vs Brain	LPGlu vs RPAla r = +0.667 p = 0.034
LPGlu vs LPAsp r = +0.673 p = 0.032	*RPGlu vs RPCys r = −0.635 p = 0.048*	*LPGlu vs RAAla r = −0.670 p = 0.034*
LPAla vs LPCys r = +0.838 p = 0.0024	RPGlu vs RHGlu r = +0.650 p = 0.041	*LPAla vs RAAla r = −0.639 p = -0.046*
LPAla vs LHCys r = +0.669 p = 0.034	*RPCys vs RHGlu r = −0.719 p = 0.018*	LPAla vs RHAsp r = +0.705 p = 0.022
LAAla vs LACys r = +0.914 p = 0.0002	RPAsp vs RAAla r = +0.650 p = 0.041	LAGlu vs RPGlu r = +0.649 p = 0.042
	RAGlu vs RAAsp r = +0.644 p = 0.044	LACys vs RHAla r = +0.640 p = 0.046
		LHGlu vs RPGlu r = +0.687 p = 0.028
		*LHGlu vs RAGlu r = −0.664 p = 0.035*
		*LHGlu vs RAAla r = −0.652 p = 0.04*
		*LHGlu vs RAAsp r = −0.684 p = 0.028*
Plasma vs Brain Left Side	Plasma vs Brain Right Side	Sham Right WKY Plasma
PlCys vs LAAla r = +0.651 p = 0.041	*PlAla vs RAAla r = −0.636 p = 0.047*	Plasma vs Plasma
		PlGlu vs PlAla r = +0.685 p = 0.028
Brain Left Side vs SBP	Brain Right Side vs SBP	PlGlu vs PlAsp r = +0.692 p = 0.026
NO CORRELATIONS	RAGlu vs SBP r = +0.650 p = 0.04	PlAla vs PlAsp r = +0.815 p = 0.004

The *p*-values and Pearson’s correlation coefficients (r) are indicated. Significant negative correlations in blue italics. Significant positive correlations in red. (L) left, (R) right, (H) hippocampus, (P) medial prefrontal cortex, (A) amygdala, (Glu) GluAP, (Ala) AlaAP, (Cys) CysAP and (Asp) AspAP.

**Table 3:** Significant intra- and inter-hemispheric correlations between brain neuropeptidase activities, between plasma aminopeptidase activities and brain neuropeptidase activities as well as between neuropeptidase activities and SBP in lesioned left WKY rats.

LESIONED LEFT WKY		
Lesioned Left WKY Left Side	Lesioned Left WKY Right Side	Lesioned Left WKY Left Side vs
		Right Side
Brain vs Brain	Brain vs Brain	*LPGlu vs RPGlu r = −0.760 p = 0.01*
LPAla vs LHAsp r = +0.688 p = 0.027	RPAsp vs RHAla r = +0.667 p = 0.034	LPGlu vs RAAsp r = +0.698 p = 0.024
*LPAsp vs LACys r = −0.871 p = 0.001*	RAGlu vs RHGlu r = +0.724 p = 0.017	LPAla vs RHCys r = +0.635 p = 0.048
*LPAsp vs LHGlu r = −0.641 p = 0.045*	RHAla vs RHCys r = +0.745 p = 0.013	LPAsp vs RPCys r = +0.714 p = 0.02
LAGlu vs LHGlu r = +0.737 p = 0.014		LPAsp vs RAGlu r = +0.796 p = 0.005
LAAla vs LACys r = +0.643 p = 0.044		*LACys vs RPCys r = −0.718 p = 0.019*
		*LACys vs RAGlu r = −0.658 p = 0.038*
		LAAsp vs RHAsp r = +0.843 p = 0.0022
		*LHAla vs RAAsp r = −0.658 p = 0.038*
		LHAla vs RHAla r = +0.775 p = 0.0084
		LHCys vs RAGlu r = +0.703 p = 0.023
		*LHCys vs RHAsp r = −0.646 p = 0.043*
		LHAsp vs RHAsp r = +0.655 p = 0.039
Plasma vs Brain Left Side	Plasma vs Brain Right Side	Lesioned Left WKY Plasma
PlAla vs LPAla r = +0.635 p = 0.048	*PlAsp vs RHAla r = −0.687 p = 0.027*	Plasma vs Plasma
PlAla vs LHAsp r = +0.843 p = 0.0021		PlGlu vs PlCys r = +0.854 p = 0.0016
*PlCys vs LAGlu r = −0.665 p = 0.035*		
*PlAsp vs LHAla r = −0.715 p = 0.019*		
Brain Left Side vs SBP	Brain Right Side vs SBP	
*LHCys vs SBP r = −0.723 p = 0.01*	*RAGlu vs SBP r = −0.688 p = 0.02*	
	RACys vs SBP *r* = +0.882 p = 0.0007	

The *p*-values and Pearson’s correlation coefficients (r) are indicated. Significant negative correlations in blue italics. Significant positive correlations in red. (L) left, (R) right, (H) hippocampus, (P) medial prefrontal cortex, (A) amygdala, (Glu) GluAP, (Ala) AlaAP, (Cys) CysAP and (Asp) AspAP.

**Table 4:** Significant intra- and inter-hemispheric correlations between brain neuropeptidase activities, between plasma aminopeptidase activities and brain neuropeptidase activities as well as between neuropeptidase activities and SBP in lesioned right WKY rats.

LESIONED RIGHT WKY		
Lesioned Right WKY Left Side	Lesioned Right WKY Right Side	Lesioned Right WKY Left Side vs
		Right Side
Brain vs Brain	Brain vs Brain	LPAla vs RAAla r = +0.662 p = 0.051
LPGlu vs LHAla r = +0.742 p = 0.021	RPAla vs RPCys r = +0.736 p = 0.023	*LPCys vs RPAsp r = −0.787 p = 0.011*
*LPAla vs LPAsp r = −0.647 p = 0.059*	RPAla vs RPAsp r = +0.741 p = 0.022	*LPAsp vs RAAla r = −0.762 p = 0.016*
	RPCys vs RACys r = +0.748 p = 0.02	LAGlu vs RPCys r = +0.741 p = 0.022
	*RAAla vs RHCys r = −0.651 p = 0.057*	LAGlu vs RACys r = +0.741 p = 0.022
	*RAAsp vs RHGlu r = −0.688 p = 0.04*	LHGlu vs RHAsp r = +0.667 p = 0.049
		*LHAsp vs RAAla r = −0.659 p = 0.053*
		LHAsp vs RHAsp r = +0.747 p = 0.02
Plasma vs Brain Left Side	Plasma vs Brain Right Side	Lesioned Right WKY Plasma
NO CORRELATIONS	*PlGlu vs RPGlu r = −0.901 p = 0.0008*	Plasma vs Plasma
	*PlGlu vs RAGlu r = −0.773 p = 0.014*	PlGlu vs PlAla r = +0.847 p = 0.003
	*PlAla vs RPGlu r = −0.690 p = 0.039*	PlGlu vs PlCys r = +0.839 p = 0.004
	*PlAla vs RAGlu r = −0.767 p = 0.015*	PlGlu vs PlAsp r = +0.817 p = 0.007
	*PlCys vs RPGlu r = −0.653 p = 0.056*	PlAla vs PlCys r = +0.986 p < 0.0001
	*PlCys vs RAGlu r = −0.802 p = 0.009*	PlAla vs PlAsp r = +0.855 p = 0.003
	*PlAsp vs RPGlu r = −0.637 p = 0.064*	PlCys vs PlAsp r = +0.871 p = 0.002
	*PlAsp vs RAGlu r = −0.745 p = 0.021*	
Brain Left Side vs SBP	Brain Right Side vs SBP	
*LHCys vs SBP r = −0.722 p = 0.02*	RPAsp vs SBP r = +0.676 p = 0.04	

The *p*-values and Pearson’s correlation coefficients (r) are indicated. Significant negative correlations in blue italics. Significant positive correlations in red. (L) left, (R) right, (H) hippocampus, (P) medial prefrontal cortex, (A) amygdala, (Glu) GluAP, (Ala) AlaAP, (Cys) CysAP and (Asp) AspAP. Correlations of *p* = 0.049 or slightly above *p* = 0.05 were included to reflect the tendency in the corresponding comparison but were not included in Figure 4 and Figure 5.
